# Supercritical Water Liquefaction of Mixed Waste Polystyrene, Polypropylene, and Polyethylene for Production of High Yield Oils

**DOI:** 10.1021/acs.energyfuels.4c01819

**Published:** 2024-07-03

**Authors:** Maria Mathew, Mohamad A. Nahil, Andrew B. Ross, Paul T. Williams

**Affiliations:** School of Chemical and Process Engineering, University of Leeds, Leeds LS2 9JT, UK

## Abstract

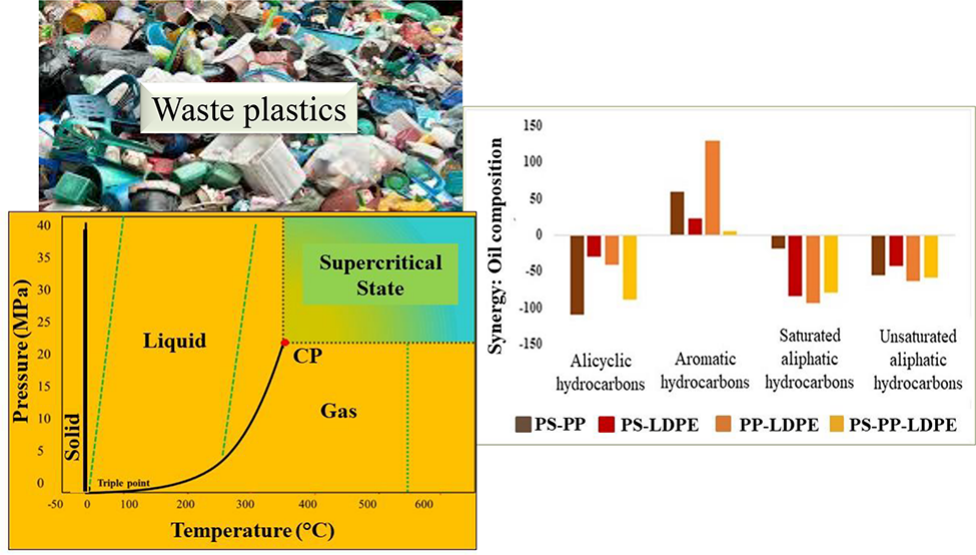

Supercritical water liquefaction of different plastic wastes has been investigated under high-temperature and high-pressure conditions. The supercritical water liquefaction of commonly used plastic types, comprising polystyrene (PS), polypropylene (PP), and low-density polyethylene (LDPE) as well as their mixtures, is reported. The experiments were carried out at varying feedstock-to-water ratios with a residence time of 60 min under supercritical water reaction conditions. The process produced high oil yields of over 97 wt %, with the highest yields obtained at a plastic:water ratio of 1:3; at higher levels of input water, the yield of oil decreased slightly. The gas phase mainly consisted of light hydrocarbons such as methane, ethane, propane, and butane, with propane found to be the most abundant gas component. Aromatic hydrocarbons and alicyclic hydrocarbons were the major products in the product oil from the supercritical water liquefaction of polystyrene and polypropylene, whereas alkanes were predominant in the oil obtained from LDPE. Analysis of the oil obtained from binary (1:1) and ternary (1:1:1) plastic mixtures showed it exhibited aromatic hydrocarbons as the major constituent, indicating synergistic interaction. It was found that the incorporation of PP in the mixture facilitated the production of cyclic compounds and suppressed the production of alkanes. Supercritical water liquefaction offers an effective solution to plastic pollution, producing valuable products without the need for catalysts.

## 1 Introduction

Global plastic production is approaching 400 million tonnes per year, with predicted production at over 3 times this value by 2060.^1^ Due to the ubiquitous use of plastics, eventually, an enormous amount of waste is also produced globally each year. According to the Organization for Economic Cooperation and Development (OECD), only about 9% of global plastic waste is recycled, whereas 50% goes into landfills, 19% is incinerated, and 22% of plastics are mismanaged and end up in different terrestrial and aquatic environments.^2^ There are different methods to manage plastic wastes, namely primary recycling, secondary/mechanical recycling, and tertiary or chemical recycling. Primary recycling is mainly applied to uncontaminated polymers that have similar properties to the virgin materials such as industrial scraps.^3^ Secondary or mechanical recycling of plastics involves utilizing mechanical methods to transform the plastics into less complex products. The typical process of secondary recycling includes cutting or shredding, separating contaminants, and floating to separate flakes of different plastics.^3^ Tertiary or chemical recycling involves transformation of plastic wastes via depolymerization into valuable chemical entities and their constituent monomers, which can be used to form new polymers or as a feedstock for petrochemicals.^4^ Tertiary thermochemical recycling methods such as pyrolysis, gasification, and hydrothermal liquefaction are gaining popularity due to their applicability to a wide range of mixed plastics.^5^

The hydrothermal liquefaction process, which includes subcritical and supercritical liquefaction, uses water at high temperature and pressure to convert waste plastics into an oil which may be used to produce liquid fuels and valuable chemicals.^6^ Boel et al.^6^ have recently reviewed the subcritical and supercritical water liquefaction of several different plastics and the influence of reaction conditions on oil yield. In addition, gaseous and solid products may also be formed. Hydrothermal liquefaction of plastics has been studied at subcritical water conditions, which typically are temperatures that range from 250 to 370 °C and pressures that maintain a liquid state. At subcritical water conditions, the properties of water change. The physicochemical properties of supercritical water, such as the dielectric constant, density, ionic product, and viscosity, significantly change from those of water at normal conditions.^7^ The density decreases and thus improves the mass transfer and solubility of organic substances in supercritical water. The reduction in density also affects various other macroscopic properties such as polarity, solvation power, degree of hydrogen bonding, viscosity, molecular diffusivity, and dielectric strength.^7^ The dielectric constant decreases sharply as temperature and pressure are increased, leading to a decrease in polarity, attributed to the weakening and disruption of hydrogen bonding between water molecules. Under supercritical water conditions, above temperatures of 374 °C and pressures of 22.1 MPa, water exhibits gas-like diffusion rates along with liquid-like collision rates so that organic compounds become highly soluble and gases are completely miscible; thereby, a single dense fluid phase is formed.^8^ Such conditions facilitate minimized mass transfer resistances and induce relatively rapid reaction rates, increased homogenization, and enhanced dissolution of organic materials, leading to the attraction of hydrothermal supercritical water liquefaction of waste plastics as a route to produce fuel oils and chemicals. The production of oils from the subcritical and supercritical water liquefaction of waste plastics is influenced by the reaction temperature and reaction time.^9−11^ For example, Chen et al.^10^ reported that increasing the temperature for the supercritical water liquefaction of polypropylene from 380 to 450 °C reduced the yield of aliphatic compounds and increased the yield of aromatic compounds in the product oil. Also, Jin et al.^11^ showed that increasing the reaction time for the supercritical water liquefaction of polyethylene increased the production of aromatic compounds in the derived oil.

Polyethylene, polypropylene, and polystyrene are among the most common types of plastic polymer types found in postconsumer waste. Supercritical water liquefaction of plastics has been investigated by various researchers, for example, for polystyrene by Kwak et al.^12^ and Musivand et al.,^13^ for polypropylene by Su et al.^14^ and Čolnik et al.,^15^ and for polyethylene by Watanabe et al.^16^ and Čolnik et al.^17^ However, the presence of multiple plastic types that are present in real-world plastic wastes poses challenges for the advancement of supercritical water liquefaction of plastic waste as a viable process. For example, the degradation behavior of plastic mixtures may be complex due to the formation of cross-reactions, thus making it difficult to predict the final oil and gas product composition based solely on the properties of the product oils and gases produced from individual plastics via supercritical water liquefaction. So, it is necessary to take into account and investigate the interactions and synergistic effects among different plastics in the waste under supercritical water liquefaction conditions. However, there have been fewer investigations of the supercritical water interaction of mixed plastics. Zhao et al.^18^ investigated the co-liquefaction of polypropylene and linear low-density polyethylene mixtures in supercritical water. The mixtures were tested at different mixing ratios ranging from 1:3 to 3:1 at 400 °C with a residence time of 60 min. They observed synergistic effects between the different plastics, which influenced the oil composition. The production of cyclic hydrocarbons and lighter hydrocarbons was promoted, while the production of alkanes was suppressed. Seshasayee and Savage^19^ investigated the hydrothermal liquefaction of an equal mixture of polypropylene, polyethylene terephthalate, polystyrene, and polycarbonate and reported that interactions between the plastics influenced the oil yield. They speculated that the synergistic effects were linked to the lowered decomposition temperature of polystyrene in the mixture, which was attributed to reactive species from the decomposition of other plastics facilitating depolymerization.

In this work, the supercritical water liquefaction of polystyrene (PS), polypropylene (PP), and low-density polyethylene (LDPE) was investigated. The plastics used were “real-world” postconsumer waste plastics produced from a commercial recycling facility. In particular, the detailed compositions of the product gases and oils were characterized in relation to supercritical water process conditions. Different mixtures of the three plastics were investigated to determine any synergistic effects of the interaction of the different plastics. Studying the specific characteristics and reactions of these plastics in supercritical water gains insights into their individual contributions to the overall process and enables optimization of the production of targeted end-products.

## 2 Materials and Methods

### 2.1 Plastics

The plastics used for the supercritical water liquefaction experiments were polystyrene, polypropylene, and low-density polyethylene, and they were obtained as recycled waste plastics from Regain Polymers Ltd. (Castleford, UK) with a particle size of approximately 2 mm pellets. A Thermos EA-2000 elemental analyzer was used for the determination of the elemental, C, H, O, N, and S analyses of the samples. A Shimadzu TGA-50 thermogravimetric analyzer (TGA) was used for the determination of proximate analysis. Table 1 shows the elemental and proximal analyses of the plastics. The proximate analysis indicated high ash content and the presence of relatively high oxygen and nitrogen content in the three plastics due to the inhomogeneity of the waste plastic samples. This may be due to incomplete full separation of the different types of plastics at the waste recycling plant, the potential for contamination of the samples by incorporation of nonplastic inert material, or the presence of additives and fillers within the plastics used in the plastic manufacturing process.

**Table 1:** Ultimate and Proximate Analyses of the Plastic Materials^a^

	ultimate analysis (wt %)				proximate analysis (wt %)			
sample	N	C	H	O	S	volatile	fixed carbon	ash
LDPE	0.37	83.17	16.34	0.12	nd	95.93	0.10	5.52
PP	0.36	82.03	16.55	1.07	nd	95.30	0.03	6.04
PS	0.42	86.09	7.87	5.63	nd	95.43	0.15	5.52

a nd = not detected.

### 2.2 Experimental Reactor System

The experimental reactor system used for the supercritical water liquefaction experiments was a 75 mL capacity Hastelloy-C autoclave reactor supplied by Parr Instrument Company Inc. (Illinois, USA). The reactor was fitted with a thermowell into which a thermocouple was inserted to measure the internal temperature of the reactor. The heating of the reactor was done via an external electrical furnace, and the temperature was monitored throughout the experiments. The pressure inside the reactor was autogenerated by heating the water to the desired temperature in the closed reactor vessel and was measured using a pressure gauge located on the top of the reactor. Additionally, a gas outlet and sampling valve were present to collect gas samples into a gas sampling syringe for further analysis. In each experiment, the volume of reactants in the reactor did not exceed 24 mL due to the limitation in pressure adjustment to attain supercritical water conditions. The plastic pellet feedstock and water were weighed and added to the reactor in different ratios (1:3, 1:4, 1:6, and 1:9) in order to study the effect of feedstock water ratio on the properties of the liquid fuel and other products. Experiments were undertaken with single plastics (PS, PP, and LDPE) and binary and ternary mixtures of the plastics. Using a closed autoclave batch reactor enabled excellent mass balance closures to be determined. The product yield data showed close to 100% mass closures. In addition, experimental reproducibility using a closed reactor system produced very good reproducibility. For example, several repeat experiments with polystyrene produced relative standard deviations for the mass balance (0.9%), the oil yield (1.1%), and the gas yield (0.5%).

The experimental procedure was as follows: after the reactor was loaded, it was sealed and purged with nitrogen for 10 min. The reactor was then heated to the designated temperature of 450 °C and pressure of 22 MPa over a period of 20 min and held at 450 °C for a further reaction time of 60 min. During the 60 min reaction period, further gases were generated, leading to an increase in pressure reaching up to 33 MPa depending on the single plastic and plastic mixture used. It should be noted that as the autoclave reactor is a batch reactor system, the reaction time also includes the heating-up and cooling-down time periods. After the experiment was completed, the reactor was removed from the furnace and cooled with compressed air to quickly cool the reactor to ambient temperature; then, the internal pressure and temperature were recorded. To determine the amount of gas product formed, the gas sampling valve was opened, allowing the gas effluent to flow into a gastight sampling syringe. The collected gas samples were then analyzed immediately using packed column gas chromatography for the identification and quantification of the gases. Following gas sampling, the reactor was opened, and the liquid and solid samples were collected separately by filtration. To recover any remaining organic oil compounds, the reactor was rinsed with a specific quantity of DCM (dichloromethane), and the resulting solution was stored in a separate container for further analysis.

### 2.3 Gas Analysis

The gas product collected in the gas sampling syringe was analyzed using different Varian 3380C gas chromatographs (GC). Each gas sample was injected into the chromatographs 3 times, and the average response was used to determine the gas yield. The analysis of permanent gases (H_2_, O_2_, N_2_, and CO) was performed using a Varian CP-3380 gas chromatograph equipped with a thermal conductivity detector (GC/TCD). The system employed a column with dimensions of 2 m in length and 2 mm in diameter, which was packed with a 60–80 mesh molecular sieve. Argon was used as the carrier gas for this gas chromatograph. The column oven was maintained at a constant temperature of 40 °C throughout the analysis, while the injector temperature was set to 120 °C. The detector temperature and filament temperature were set to 120 and 160 °C, respectively. Carbon dioxide analysis was conducted using a separate Varian CP-3380 (GC/TCD) instrument with a column packed with a Hysep 80–100 molecular mesh, and argon was used as the carrier gas. Regular calibration of the gas chromatographs was performed using a standard gas mixture, which consisted of 1% H_2_, O_2_, CO, and CO_2_ and 96% N_2_ in volume percentages.

To analyze hydrocarbon gases, a different Varian CP-3380 gas chromatograph equipped with a flame ionization detector (GC/FID) was used. The GC was equipped with a column (2 m in length and 2 mm in diameter) packed with Hysep 80–100 mesh. Nitrogen was used as the carrier gas. The injector temperature was maintained at 150 °C, while the detector temperature was set at 200 °C. The oven temperature program started at 60 °C for 3 min, followed by heating to 100 °C at a rate of 10 °C/min. It was then held at 100 °C for 3 min before ramping up to 120 °C at a rate of 20 °C/min. For calibration, the GC was regularly calibrated by using a standard gas mixture. The standard gas mixture for alkanes contained 1% volume CH_4_, C_2_H_6_, C_3_H_8_, and C_4_H_10_ with the remaining volume consisting of N_2_. Similarly, for alkenes, a mixture of hydrocarbon gases containing 1% volume C_2_H_4_, C_3_H_6_, C_4_H_8_, and C_4_H_10_ with N_2_ as the makeup gas was used for calibration.

The product gases were analyzed for volume concentration, molar concentration, volume percentage, and mass percentage. Response factors (RFs) for each species in the standard gases identified the gases produced and were used to calculate the volume percentages of each gas. The mole numbers of each gas were calculated using the ideal gas law, which allowed for the determination of gas yields in terms of moles of gas per gram of each plastic feedstock. Therefore, the GC peak area of each gas compound was used to calculate the volume and mass percentages as well as the molar concentration.

### 2.4 Oil Analysis

Gas chromatography–mass spectrometry was used to analyze in detail the composition of the product oil using a Varian 3800-GC instrument coupled with a Varian Saturn 2200 ion trap detector system operating in tandem mass spectrometry mode (GC-MS/MS system). A 5000 ppm (parts per million) oil sample was prepared in DCM. A GC Varian VF-5 ms (DB-5 equivalent) capillary column with dimensions of 30 m in length and 0.25 mm in inner diameter was employed for the separation of the compounds. Helium gas was used as the carrier gas with a constant flow rate of 1 mL/min. The GC injector was maintained at a temperature of 290 °C. The oven temperature program consisted of an initial hold at 40 °C for 2 min, followed by a ramp up to 280 °C at a rate of 5 °C/min. The temperature was then held at 280 °C for 10 min. The transfer line temperature to the MS/MS ion trap system was set to 280 °C, and the trap temperature was held at 200 °C. To identify and quantify the oil components, the analysis made use of the National Institute of Science and Technology (NIST) compound library, which contains information about various compounds. For aliphatic compounds, a standard mixture of C8–C40 compounds (500 ppm) obtained from Sigma-Aldrich UK was employed as reference for identification and quantification. Additionally, a set of standard aromatic and oxygenated compounds was also employed as a reference.

## 3 Results and Discussion

### 3.1 Supercritical Water Liquefaction of Individual Plastics

The individual plastics (PS, PP, and LDPE) underwent supercritical water liquefaction at different plastic-to-water ratios of 1:3, 1:4, 1:6, and 1:9 at a temperature of 450 °C, a final pressure of 33 MPa, and a reaction time of 60 min. The gaseous products were analyzed using packed column gas chromatography, and the oil product composition was analyzed using capillary column gas chromatography–mass spectrometry.

#### 3.1.1 Product Yield

Table 2 shows the yields of gas, oil, and solid product from the supercritical water liquefaction of polystyrene, polypropylene, and low-density polyethylene. The solid product from the different plastics was negligible in all cases. The overall mass balance was close to 100.0 wt % mass closure, reflecting the excellent reproducibility of the experimental closed batch autoclave reactor system used. The results show that oil is the major product irrespective of the plastic type investigated. The oil yield results are expressed in terms of the mass of input plastic and input reacted water. In the supercritical hydrothermal liquefaction of plastics, water is regarded as a reactant, and therefore, water was included in the percentage yield calculation. For example, experiments using deuterium oxide (D_2_O) instead of H_2_O for reactions of organic materials in supercritical water have demonstrated that the hydrogen in the reaction products such as methane (as CD_4_) and hydrogen gas (D_2_) originates from the water.^20,21^ Therefore, incorporating water as a reactant in the percentage yield calculation offers a more accurate representation of the overall reaction yield and mechanism in the supercritical hydrothermal liquefaction of plastics. High oil yields were obtained at all of the plastic:water ratios used between 1:3 and 1:9, with the highest yield for polystyrene at 98.2 wt %, for polypropylene at 97.5 wt %, and for low-density polyethylene at 98.0 wt %. There was a slight reduction in oil yield and slight increase in gas yield as the plastic:water ratio was changed from 1:3 to 1:9, that is, as the amount of plastic in the reactor was reduced. According to Bai et al.,^22^ increasing the feedstock concentration within a certain range enhances the conversion of plastic into desirable products, whereas exceeding the hydrolysis capacity threshold leads to a decrease in liquefaction rate. Yan et al.^23^ reported that the oil yield from polystyrene is inhibited by the stability of the benzene ring in the polystyrene structure preventing easy depolymerization. However, the oil yields obtained for polystyrene in this work are very similar to those from the polyalkene plastics (PP and LDPE), which may be due to the long residence time in the supercritical water regime (60 min in this work) enabling fuller depolymerization of the polystyrene. This is supported by the increase in gas yield and the noted increase in reactor pressure due to the production of gas over the 60 min reaction time period. The yields of oil are similar to those reported in the literature, for example, those reported by Kwak et al.,^12^ who researched the hydrothermal liquefaction of polystyrene under subcritical and supercritical water conditions at temperatures and pressures of 370–420 °C and 24–32 MPa. Under subcritical water conditions, conversion to oil was ∼80%, but under supercritical water conditions, conversion was ∼100%. Chen et al.^10^ investigated the supercritical water liquefaction of polypropylene and reported an oil yield of 91 wt % at a temperature of 450 °C and a pressure of 23 MPa with a 1 h reaction time. Su et al.^24^ reported an oil yield of ∼92 wt % for the supercritical water liquefaction of polyethylene at conditions of 460 °C and a water:plastic ratio of 6:1. Jin et al.^11^ obtained a maximum oil yield of 87% from the supercritical water liquefaction of polyethylene at a temperature of 450 °C and a reaction time of 45 min. Seshasayee and Savage^25^ investigated the effect of holding time and temperature on the product oil yield from the supercritical water liquefaction of polypropylene, polystyrene, polycarbonate, and polyethylene terephthalate (PET). The highest oil yields for each plastic ranged from 16 wt % for PET to 86 wt % for polystyrene.

**Table 2:** Product Gas and Oil Yields (wt %) from the Supercritical Water Liquefaction of Different Plastics in Relation to Plastic:Water Ratios at Conditions of 450 °C and Final Pressure of 33 MPa with a Residence Time of 60 min

	plastic:water ratio			
	1:3 (wt %)	1:4 (wt %)	1:6 (wt %)	1:9 (wt %)
Polystyrene				
gas yield	1.2	1.2	1.3	1.3
oil yield	97.6	98.2	98.2	98.2
solid	1.4	0.6	0.5	0.5
mass balance	100.2	100.0	100.0	100.0
Polypropylene				
gas yield	2.2	2.2	2.3	2.6
oil yield	97.4	97.5	97.4	97.2
solid	0.5	0.3	0.3	0.2
mass balance	99.9	100.0	100.0	100.0
Low-Density Polyethylene				
gas yield	2.0	2.2	2.3	2.7
oil yield	98.0	97.8	97.7	97.3
solid	<0.1	<0.1	<0.1	<0.1
mass balance	100.1	100.1	100.1	100.1

#### 3.1.2 Gas Composition

**Figure 1:**
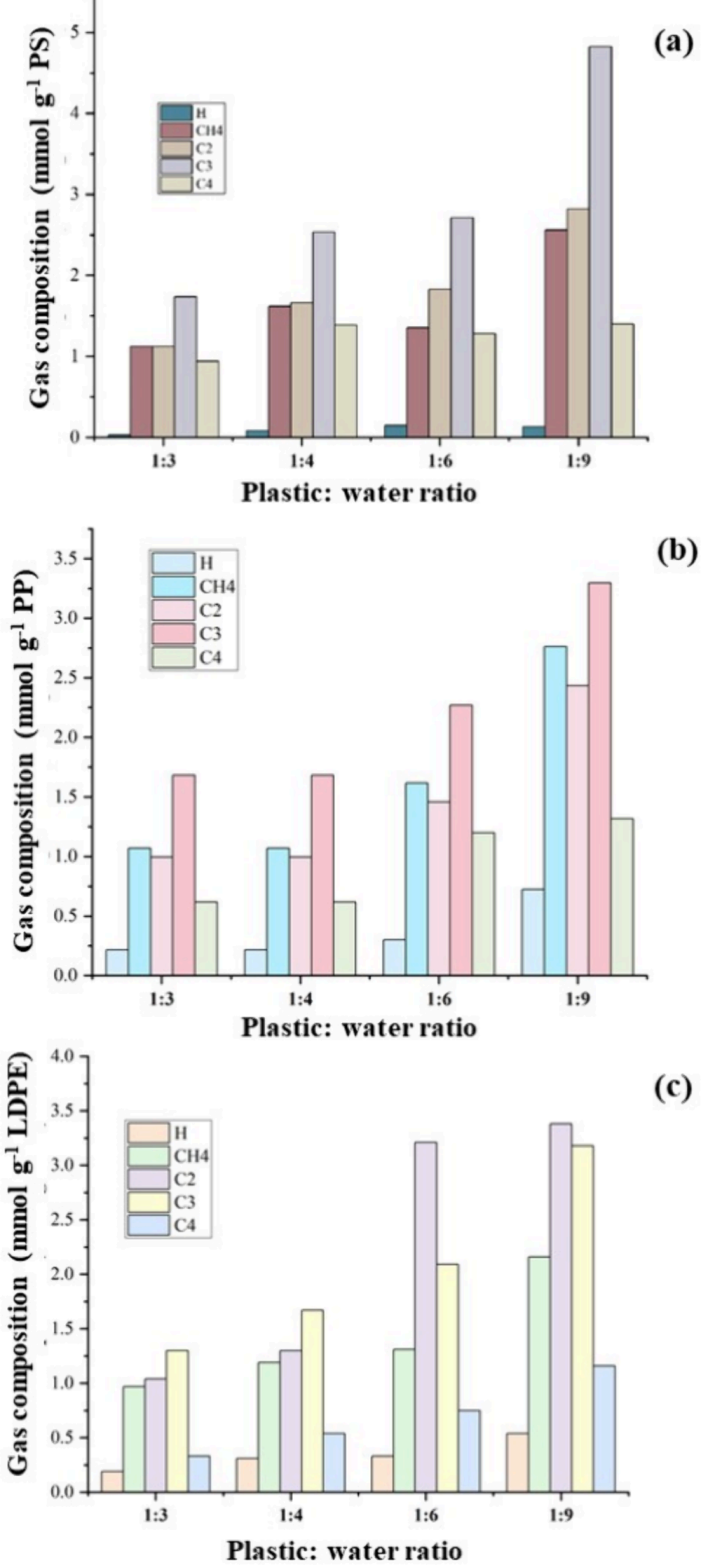
Composition of the gas produced from the supercritical water liquefaction of (a) polystyrene, (b) polypropylene, and (c) low-density polyethylene in relation to the plastic:water ratio at conditions of 450 °C and final pressure of 33 MPa with a residence time of 60 min.

Figure 1 shows the composition for the product gas produced from the supercritical water liquefaction of polystyrene, polypropylene, and low-density polyethylene in relation to the plastic:water ratio. For polystyrene, the total gas yield for the different plastic:water ratios was between 1.2 and 1.3 wt % (Table 2). Figure 1a shows that the main gases produced from polystyrene were hydrocarbons consisting of alkanes, methane, ethane, propane, and butane and also smaller quantities of the alkene gases ethene, propene, and butene, which are similar to the results reported by Bai et al.^22^ and Liu et al.^26^ Propane yields were notably higher compared to the other hydrocarbon gases. In regard to the supercritical water liquefaction of polypropylene, the gaseous product was mainly composed of propane, propene, ethane, methane, and butane (Figure 1b). It may also be observed that the C3 gas components, propane and propene, were the dominant gas products, with the majority being propane. Propane is produced through an endothermic reaction involving the decomposition of oily components. It has been observed that prolonged reaction times and elevated temperatures lead to a notable rise in propane concentration due to the hydrogenation of propene.^15^ The increased propane concentration may also be attributed to the increased temperature and longer residence time used for the experiment that facilitated the hydrogenation of propene. For low-density polyethylene (Figure 1c), the highest gas yield was achieved at a feedstock-to-water ratio of 1:9. All of the individual hydrocarbon gases produced from the supercritical water liquefaction of the three plastics showed an increase as the plastic:water ratio increased. Previous work on the hydrothermal liquefaction of polyethylene by Zhang et al.^27^ and Su et al.^24^ also showed that longer residence time and higher temperature such as 450 °C result in increased gas production.

The yield of methane and hydrogen in the gaseous products may also be due to the contribution of hydrogen atoms from the supercritical water.^19^ In the work reported here, the nature of the reactive supercritical water will be involved in the chemical degradation of the plastics. The role of water in supercritical water liquefaction processes is multifaceted, encompassing functions as a solvent, reactant, and catalyst.^28^ Unlike normal water, supercritical water is completely miscible with nonpolar gases and organic compounds due to its low density, which significantly alters interactions between water molecules and ions. As temperature increases, the ion product of water initially rises, reaching a maximum (>10–11 mol^2^/L^2^) at around 350 °C and 30 MPa. This increased ion concentration promotes acid–base-catalyzed reactions such as hydrolysis. However, with further temperature increases and reduced density, the ion product plunges (∼10–21.6 mol^2^/L^2^ at 450 °C and 25 MPa), favoring free radical mechanisms.^29^ The special properties of supercritical water near the critical point, including changes in solvation and ionic dissociation, significantly impact the reaction mechanisms. For instance, the solvation effect in supercritical water can increase reaction rates by several orders of magnitude,^30^ while the dissociation activation energy barrier for H_2_O_2_ is reduced compared to gas-phase conditions.^31^ Near the critical point, the dissociation constant (*K*_w_) of water is much higher, facilitating acid-catalyzed reactions without added acids. However, above the critical point, *K*_w_ drops sharply, making ionic reactions less significant and shifting dominance to free radical mechanisms, particularly in the presence of oxidants.^32,33^ Self-dissociation of water into hydroxyl ions and hydrogen ions facilitates a free radical reaction mechanism in which the radicals interact with the organic compounds, leading to bond scission and the formation of new products.

It has been reported that under supercritical water conditions, intermolecular and intramolecular hydrogen bonding become disrupted, making hydrogen available for chemical reactions.^20,34^ Park and Tomiyasu^21^ substituted D_2_O as the reaction medium instead of H_2_O for the supercritical water gasification of organic compounds with a catalyst (Ru_2_O) and reported that hydrogen gas and the hydrogen contained in methane were produced from the water rather than from the degradation of the organic compounds. The ability of water to provide hydrogen during the supercritical hydrothermal liquefaction process is crucial, as it affects the nature of the products formed. Hydrogenation plays a key role in the termination of chain-forming free radical reactions. Therefore, the release of hydrogen from water can terminate the reaction, resulting in the production of numerous small organic molecules with low molecular weight.^35^ Kruse et al.^34^ also concluded that water molecules release hydrogen atoms, facilitating the intramolecular bond rupture of reactants.

Supercritical water conditions generate high concentrations of H^•^ and OH^•^ radicals from the water that are involved in the depolymerization of the plastic polymer.^36^ Additionally, H^•^ and OH^•^ can be involved in acid–base-type catalytic reactions.^37^ For example, Guo et al.^35^ reported that the ionization of water at high temperatures allows it to function as an effective acid catalyst^35^ or as a base catalyst.^38^

Free radical mechanisms also play a significant role in supercritical water reactions, and the factors that influence free radical formation are temperature, the presence of catalysts, and the type of reactants.^39^ Additionally, water density has also been reported to affect free radical mechanisms. Henrikson et al.^40^ noted that different water densities can either accelerate or inhibit supercritical water reactions at the same temperature. The influence of water on the reaction depends on the mechanism involved; at high water density, water can accelerate ionic reaction mechanisms, while at low water density, it favors free radical mechanisms. At high temperatures and pressures in supercritical water, aliphatic hydrocarbons can experience rapid decomposition and degradation. The reaction behavior of aliphatic hydrocarbons in supercritical water is primarily dominated by free radical mechanisms, leading to the cleavage of carbon–carbon bonds and the formation of smaller organic fragments.

#### 3.1.3 Oil Composition

**Figure 2:**
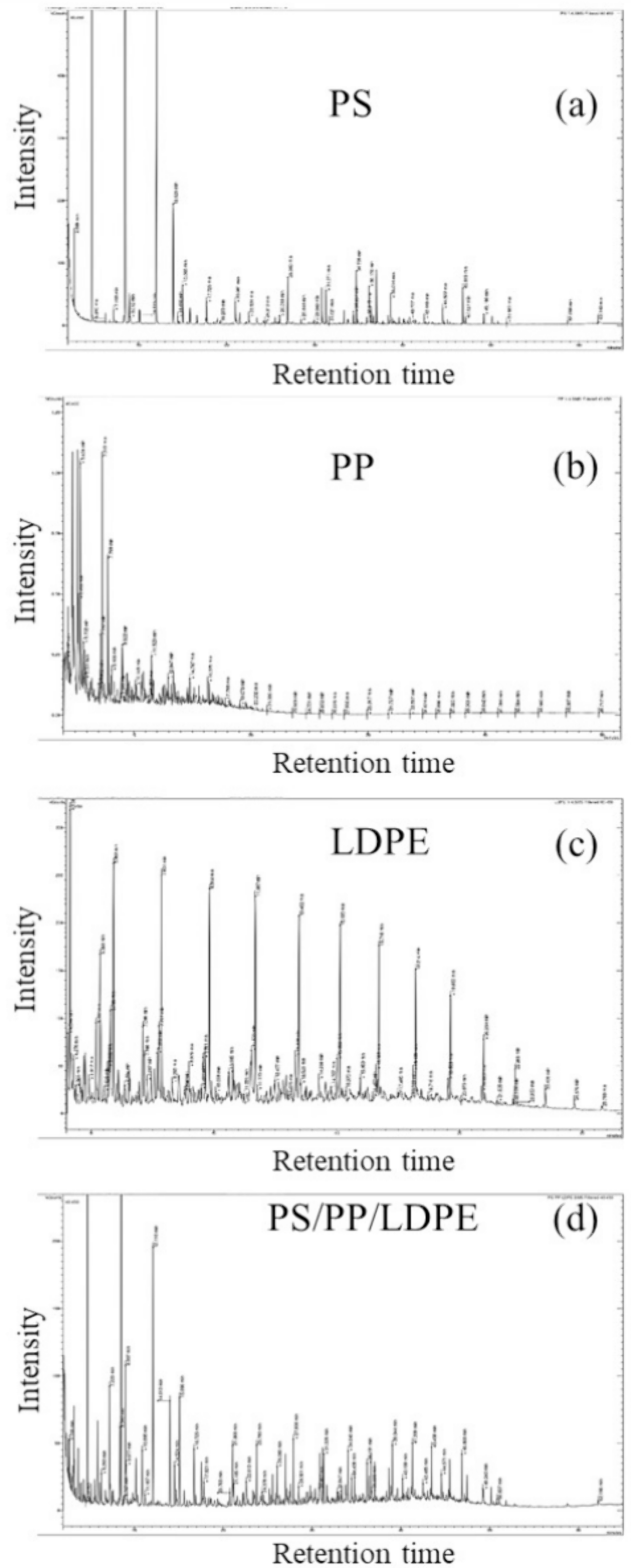
GC-MS/MS total ion chromatograms for oils derived from the supercritical water liquefaction of (a) polystyrene, (b) polypropylene, (c) low-density polyethylene, and (d) the mixture of the three plastics at conditions of 450 °C and final pressure of 33 MPa with a residence time of 60 min and plastic:water ratio of 1:4.

Figure 2a–c shows the GC-MS/MS total ion chromatograms (TICs) for oils derived from the supercritical water liquefaction of polystyrene, polypropylene, and low-density polyethylene. In addition, the GC-MS/MS TIC for the 1:1:1 mixture of the three plastics is shown in Figure 2d. The process conditions were a temperature of 450 °C and a final pressure of 33 MPa with a residence time of 60 min and a plastic:water ratio of 1:4. The TIC for polystyrene indicates a mix of mainly single and polycyclic aromatic compounds produced from the degradation of the aromatic polymer structure. The ion chromatogram for polypropylene indicates a range of hydrocarbons was formed, including aliphatic hydrocarbons, cyclic hydrocarbons, and aromatic hydrocarbons. The oil produced from the supercritical water liquefaction of polyethylene (LDPE) shows the regular carbon number distribution of alkane and alkene hydrocarbons derived from the scission of the LDPE polymer. The major peak for each carbon number was the alkane, with subsidiary concentrations of alkene and alkadiene hydrocarbons of the same carbon number. The oil composition results align with the results reported by Seshasayee and Savage^25^ for the supercritical hydrothermal liquefaction of polystyrene and polypropylene at a temperature of 450 °C and a pressure of 25 MPa. They reported that polystyrene decomposed to give oil that was rich in aromatic content, whereas the oil from polypropylene consisted of cycloalkanes and aromatic polycyclic compounds.

Figure 2d shows the TIC for the mixture of the three plastics, showing the contribution of aromatic compounds particularly at lower retention times (<20 min) from the polystyrene and polypropylene pyrolysis and also aliphatic compounds at higher retention times (>20 min), particularly the regular series of alkanes, alkenes, and alkadienes. However, section 3.3 later shows that synergistic effects are demonstrated due to the interaction of the three plastics.

##### 3.1.3.1 Polystyrene

Table 3 shows the detailed analysis of the individual identified compounds found in the product oil derived from the supercritical water liquefaction of polystyrene, representing approximately 30 of the highest concentration compounds. The total peak area of those identified compounds was 96.245%, illustrating that almost all of the compounds present in the oil were identified, with the remaining 3.755% representing low concentration compounds.

**Table 3:** Compounds Identified by GC-MS/MS in the Product Oil from the Supercritical Water Liquefaction of Polystyrene at Conditions of 450 °C and Final Pressure of 33 MPa with a Residence Time of 60 min and Plastic:Water Ratio of 1:4^a^

retention time (min)	peak area (%)	compound	concentration (mg g^–1^ of PS)
2.685	0.932	benzene	7.94
4.709	26.399	toluene	224.94
7.162	0.29	cyclohexane, 1-ethyl-1,4-dim	2.47
8.534	38.447	ethylbenzene	327.60
8.933	0.587	*p*-xylene	5.00
10.092	0.461	styrene	3.93
12.055	12.868	benzene, (1-methylethyl)-/cumene	109.65
13.928	3.544	benzene, propyl-	30.20
14.441	0.214	benzene, 1-ethyl-2-methyl-	1.82
15.004	1.154	benzene, 1,2,3-trimethyl-	9.83
15.837	0.502	α-methylstyrene	4.28
16.633	0.266	benzene, 1,2,4-trimethyl-	2.27
17.726	0.693	benzene, (1-methylpropyl)-	5.90
18.947	0.2	benzene, 2-propenyl-	1.70
20.982	0.643	benzene, butyl-	5.48
26.944	1.054	naphthalene	8.98
30.811	0.736	2-methylnaphthalene	6.27
31.272	0.741	1*H*-indene, 1-ethylidene-	6.31
33.314	0.262	biphenyl	2.23
34.387	0.252	naphthalene, 1,4-dimethyl-	2.15
34.728	1.046	diphenylmethane	8.91
36.176	1.187	benzene, 1,1′-ethylidenebis-	10.11
36.384	0.341	1,1′-biphenyl, 4-methyl-	2.91
36.647	0.311	4,4′-dimethylbiphenyl	2.65
37.002	1.04	bibenzyl	8.86
38.614	0.504	3,3′-dimethylbiphenyl	4.29
46.82	0.81	2-phenylnapthalene	6.90
49.198	0.236	naphthalene, 2-(phenylmethyl)	2.01
50.123	0.214	*m*-terphenyl	1.82
62.148	0.311	1,3,5-triphenylbenzene	2.65
total	96.245		820.09

a Listed are the highest concentration compounds identified in the oil.

Table 3 shows the chemical composition of the major components of the oil produced from the supercritical hydrothermal liquefaction of polystyrene. It can be observed that benzene derivatives form more than 90% of the total composition, followed by naphthalene derivatives. Bai et al.^22^ reported that the main components in the oil derived from the supercritical water liquefaction of polystyrene consisted of monoaromatic compounds and polycyclic aromatic compounds. The oil composition obtained by GC analysis was consistent with the results reported by Kwak et al.,^12^ where the major components of the oil were benzene derivatives such as ethylbenzene, toluene, and cumene and naphthalene derivatives such as phenylnaphthalene and methylnaphthalene. It can be noticed that the selectivity for styrene and α-methylstyrene produced is low in this supercritical water liquefaction process compared to thermal pyrolysis processes, where styrene is the major product. This can be attributed to the degradation of styrene trimers, dimers, and monomers at longer residence times under supercritical water conditions, leading to the production of ethylbenzene, toluene, and isopropyl benzene.^12^ The results for the supercritical water liquefaction of polystyrene may be compared to those previously reported in the literature. For example, Kwak et al.^12^ studied the depolymerization of polystyrene in near- and supercritical water. The experiments were carried out under reaction conditions with temperatures of 370–420 °C and pressures between 24 and 32 MPa in an autoclave reactor. A shift in the selectivity of products was observed at 400 °C and 28 MPa, where the selectivity for styrene monomers, dimers, and trimers decreased, while the selectivity for toluene, ethylbenzene, and isopropyl benzene increased. Musivand et al.^13^ reported on the supercritical water recycling of polystyrene using a stainless-steel microreactor at temperatures ranging from 300 to 360 °C with autogenerated pressures; holding times ranged from 1 to 4 h. Complete decomposition of polystyrene into oil (83%) and water-soluble compounds (10%) was achieved at 360 °C and a reaction time of 4 h. The liquid oil consisted of aromatic compounds (1–3 aromatic rings) with a low quantity of styrene, while the water phase contained both aromatic and oxygenated compounds such as benzaldehyde and acetophenone. Su et al.^24^ reported on the supercritical water liquefaction of polystyrene and the influence of various reaction parameters such as reaction temperature, pressure, and water-to-plastic ratio. They showed that as the temperature and residence time were increased, the yield of the gaseous products increased while the oil yield was reduced. The product yield as well as its chemical composition was influenced by the water:plastic ratio, where a ratio of 6:1 was found to give >90% oil yield.

Yan et al.^23^ have proposed, based on molecular dynamics modeling and density functional theory, that the degradation of polystyrene under supercritical water conditions involves an initial production of styrene oligomers. Further depolymerization of the oligomers yields monoaromatic hydrocarbons such as benzene, toluene, styrene, and ethylbenzene as well as polycyclic aromatic hydrocarbons such as naphthalene and biphenyl. Short-chain alcohols may also form via the radicals produced in supercritical water.

**Figure 3:**
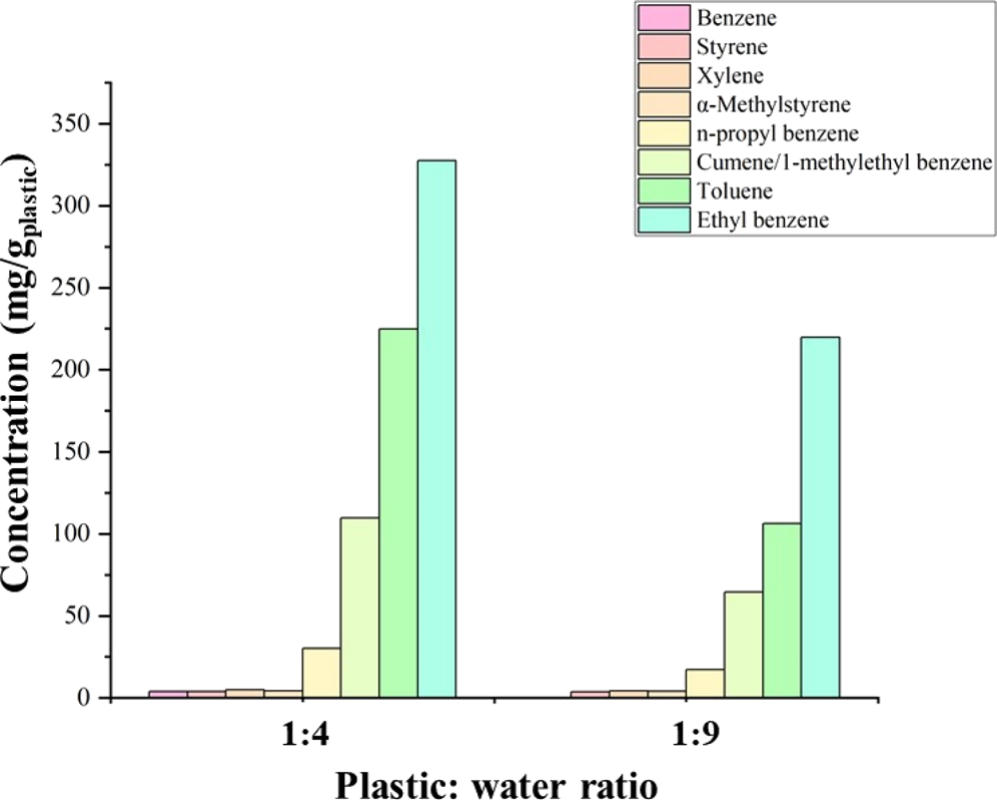
Composition of the oil produced from the supercritical water liquefaction of polystyrene in relation to plastic:water ratios of 1:4 and 1:9 at conditions of 450 °C and final pressure of 33 MPa with a residence time of 60 min.

Figure 3 shows the influence of changing the plastic:water ratio on the concentration of the main components identified in the product oil from the supercritical water liquefaction of polystyrene. Changing the plastic:water ratio from 1:4 to 1:9 resulted in a clear reduction in the concentration of aromatic compounds in the product oil.

##### 3.1.3.2 Polypropylene

Table 4 shows the concentration of the compounds identified in the product oil derived from the supercritical water liquefaction of polypropylene, representing approximately 30 of those with the highest concentration. The reported total peak area of the identified compounds was 65.459% of the total compounds in the oil, the remaining percentage being the compounds at low concentration. There was a high proportion of low molecular weight compounds present in the oil, reflected in the lower retention times of the species identified. Cyclic and aromatic compounds dominate the oil composition, including cyclic alkanes and alkenes and alkylated benzenes.

**Table 4:** Compounds Identified by GC-MS/MS in the Product Oil from the Supercritical Water Liquefaction of Polypropylene at Conditions of 450 °C and Final Pressure of 33 MPa with a Residence Time of 60 min and Plastic:Water Ratio of 1:4^a^

retention time (min)	peak area (%)	compound	concentration (mg g^–1^ of PP)
2.085	0.99	cyclohexane	11.80
2.197	1.718	2-pentene, 4-methyl-	20.47
2.59	0.878	2-pentene, 2,4-dimethyl-	10.46
3.039	1.392	1,3-dimethylcyclopentane	16.59
3.258	1.538	3-methylhexene	18.33
3.694	1.059	1,1,3-trimethylcyclopentane	12.62
4.057	0.993	3-methyleneheptane	11.83
4.725	4.678	toluene	55.75
5.17	0.775	1,3-dimethylcyclohexane	9.24
5.882	1.31	2,4-dimethylhexane	15.61
6.134	1.246	cyclopentane, 1,1-ethylidene/cyclohexane, 1-ethyl-2-methyl	14.85
6.302	2.477	cyclopentane, 1,1,3,4-tetramethyl	29.52
6.754	1.076	3-ethylhexane	12.82
7.238	9.848	cyclohexane, 1,1,3-trimethyl	117.36
7.506	0.926	cyclohexane, 1-ethyl-1,3-dimethyl	11.03
7.623	2.13	6-methyl-1-octene	25.38
8.396	7.261	cyclohexane, 1,1,4-trimethyl	86.53
8.536	0.806	ethylbenzene	9.60
8.806	0.747	nonene	8.90
9.012	7.444	*p*-xylene	88.71
9.873	1.291	cyclohexane, 1,1,3,5-tetramethyl	15.38
10.063	0.72	1,2,3-cyclohexane	8.58
10.213	1.022	*o*-xylene	12.18
10.836	1.12	cyclopentane, 1-methyl-3(2-methyl-1-propenyl)	13.35
14.556	1.157	1-ethyl-3-methylbenzene	13.79
15.13	5.052	benzene, 1,2,3-trimethyl	60.20
16.759	2.738	benzene, 1,2,4-trimethyl	32.63
18.342	0.707	3,5-dimethyloctane	8.43
21.216	0.87	benzene, 4-ethyl-1,2-dimethyl	10.37
21.444	0.71	2,2-dimethyloctene	8.46
21.612	0.78	2-butyl-3-methyl-1-pentene	9.30
total	65.459		780.05

a Listed are the highest concentration compounds identified in the oil.

**Figure 4:**
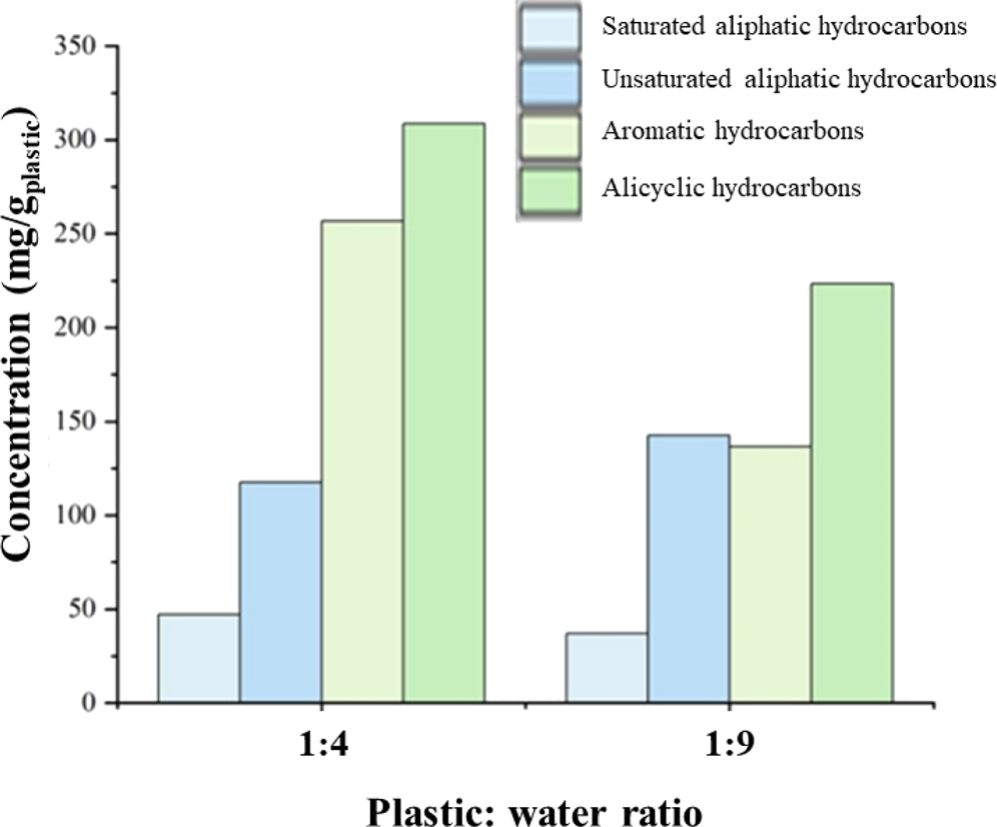
Composition of the oil produced from the supercritical water liquefaction of polypropylene in relation to plastic:water ratios of 1:4 and 1:9 at conditions of 450 °C and final pressure of 33 MPa with a residence time of 60 min.

Figure 4 shows the chemical class composition of the product oil from the supercritical water liquefaction of polypropylene in relation to plastic:water ratios of 1:4 and 1:9. The results show that the chemical composition of the oil was influenced by the reaction conditions. The oil was mainly composed of the primary chemical groups saturated and unsaturated aliphatic hydrocarbons, alicyclic hydrocarbons, and aromatic hydrocarbons, with the alicyclic hydrocarbons and aromatic compounds forming the majority of the total oil composition. The alicyclic hydrocarbons were predominantly formed due to α-alkenes derived from polypropylene undergoing cyclization reactions. Cyclic hydrocarbons and unsaturated aliphatic compounds (alkenes) have identical chemical formulas when compared to those of saturated aliphatic compounds (alkanes) and aromatics. Consequently, cyclization was given preference over saturation and aromatization in the reaction, which explains the increased concentration of alicyclic compounds.^10^ Moreover, in the molecular structure of polypropylene, a significant number of tertiary carbons are present. Compared with secondary carbons, tertiary carbons provided a greater opportunity for oligomers to produce cyclic hydrocarbons. As a result, a higher cyclic content was observed in the oil obtained from the supercritical water liquefaction of polypropylene. On the other hand, the aromatic compounds likely came from cyclic hydrocarbons undergoing dehydrogenation reactions. Among the aromatic compounds identified, xylene and mesitylene (1,3,5-trimethylbenzene) were the most abundant, whereas cyclohexane derivatives such as 1,1,3-trimethylcyclohexane predominated among the alicyclic compounds. Previous studies show that increased temperature also facilitates the production of cyclic and aromatic compounds.^15^ Su et al.^14^ reported that the main components produced in the oil from the supercritical water liquefaction of polypropylene were *n*-alkanes and *n*-alkenes.

The supercritical water liquefaction of polypropylene has also been reported in the literature. For example, the liquefaction of polypropylene using supercritical water was studied by Chen et al.^10^ They reported a maximum oil yield of 90–91% at 450 °C within the time period of 0.5–1 h; alkanes, alkenes, cyclic compounds, and aromatic hydrocarbons were the major components of the oil product. Su et al.^14^ investigated the impact of variations in pressure and temperature on the degradation reactions of polypropylene in supercritical water and concluded that the temperature and pressure conditions have a significant role in determining the formation of the end-products. Čolnik et al.^15^ investigated the supercritical liquefaction of polypropylene in a pressure batch reactor at temperatures of 425 and 450 °C with different holding times between 15 and 240 min. The results showed oil yields of 95% consisting of alkanes, alkenes, cycloalkanes, and alcohols. The gas yield was about 20% and was composed of light hydrocarbons (C1–C6) with propane as the most abundant gas component.

##### 3.1.3.3 Low-Density Polyethylene

Table 5 shows the compounds present in the oil derived from the supercritical water liquefaction of low-density polyethylene, representing approximately 30 of the highest concentration compounds. The identified compounds in Table 5 represent 70.284% of all the oil compounds, the remaining percentage representing compounds in low concentration. The product oil was mainly composed of aliphatic hydrocarbons, particularly the series of *n*-alkanes, which can also be observed in the GC-MS/MS TIC in Figure 2c. Also present in the oil were low molecular weight aromatic compounds such as ethylbenzene and xylenes.

**Table 5:** Compounds Identified by GC-MS/MS in the Product Oil from the Supercritical Water Liquefaction of Low-Density Polyethylene at Conditions of 450 °C and Final Pressure of 33 MPa with a Residence Time of 60 min and Plastic:Water Ratio of 1:4^a^

retention time (min)	peak area (%)	compound	concentration (mg g^–1^ of LDPE)
4.044	2.105	1-ethyl-3-methylcyclopentane	16.58
4.138	5.67	C8 octane	44.65
4.181	1.173	1,3-dimethylcyclohexane	9.24
4.277	1.478	1,4-pentadiene, 2,3,4-trimethyl	11.64
4.701	1.163	1,2-dimethylcyclohexane	9.16
4.757	0.883	2-methylethyl cyclohexane	6.95
5.197	1.362	ethylbenzene	10.73
5.359	4.299	*p*-xylene	33.85
5.66	0.861	nonadiene	6.78
5.742	1.022	cyclohexene, 3,3,5-trimethyl	8.05
5.781	1.956	*o*-xylene	15.40
5.908	4.756	*n*-nonane C9	37.45
7.099	1.711	1-ethyl-4-methylbenzene	13.47
7.166	0.98	benzene, 1-ethyl-2-methyl-	7.72
7.689	1.148	decene/decadiene	9.04
7.757	1.769	decene/1,3,5-trimethylbenzene	13.93
7.862	4.187	*n*-decane C10	32.97
8.554	1.402	benzene, 2-propenyl/indane	11.04
8.978	0.973	benzene, 1,4-diethyl-	7.66
9.56	1.607	benzene, 2-propenyl/indane	12.66
9.648	1.307	undecane	10.29
9.813	4.373	*n*-undecane C11	34.44
11.529	1.536	dodecane	12.10
11.686	4.448	*n*-dodecane C12	35.03
13.318	1.134	tridecane	8.93
13.462	3.866	*n*-tridecane C13	30.44
15.01	1.099	7-tetradecene	8.65
15.136	3.186	*n*-tetradecane C14	25.09
16.718	2.977	*n*-pentadecane C15	23.44
18.214	2.483	*n*-hexadecane C16	19.55
19.635	1.99	*n*-heptadecane C17	15.67
20.983	1.38	*n*-octadecane C18	10.87
total	70.284		553.49

a Listed are the highest concentration compounds identified in the oil.

**Figure 5:**
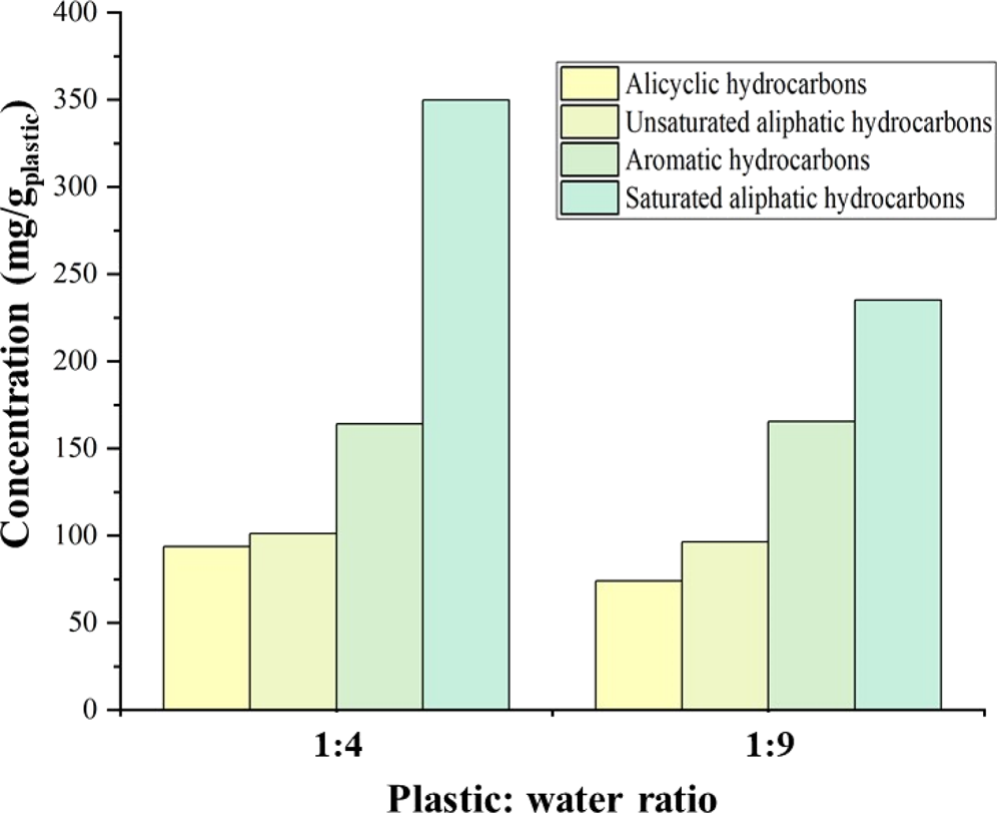
Composition of the oil produced from the supercritical water liquefaction of low-density polyethylene in relation to plastic:water ratios of 1:4 and 1:9 at conditions of 450 °C and final pressure of 33 MPa with a residence time of 60 min.

GC-MS/MS analysis of the product oil from the supercritical water liquefaction of low-density polyethylene in relation to different plastic:water ratios of 1:4 and 1:9 was carried out. The compounds identified in the oil were categorized into saturated aliphatic hydrocarbons, unsaturated aliphatic hydrocarbons, alicyclic hydrocarbons, and aromatic hydrocarbons, and the results are shown in Figure 5 for the oil produced at plastic:water ratios of 1:4 and 1:9. The results show that saturated aliphatic hydrocarbons formed the major composition of the oil phase, followed by aromatic hydrocarbons. The supercritical water liquefaction of polyethylene has been investigated and reported by Watanabe et al.^41^ at temperatures ranging from 400 to 500 °C, pressures between 20 and 40 MPa, and a residence time of 30 min. It was found that changing the temperature and pressure under supercritical water conditions significantly altered the water density, which in turn had an impact on the product distribution, particularly increasing the yield of alkanes at higher water densities (higher pressures). They reported that the product oil contained high yields of shorter-chain hydrocarbons, a higher 1-alkene/*n*-alkane ratio, and higher conversion was obtained. The supercritical water degradation of low-density polyethylene has also been investigated by Čolnik et al.^17^ The oil product was composed of alkanes, alkenes, cycloalkanes, aromatics, and alcohols. For example, higher temperatures produced a decrease in the concentration of larger (>C20) hydrocarbons and an increase in the concentration of short-chain hydrocarbons (C6–C8). The production of aromatic compounds was found to increase at higher temperatures and longer residence times.

The high production of alkanes found in the product oil from the supercritical water liquefaction of polyethylene in this work can be attributed to the structure of polyethylene with less branching that leads to random bond scission along the main chain, resulting in the production of straight-chain oligomers with varying lengths.
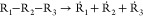


Unsaturated hydrocarbons played a significant role in determining the distribution of these oligomers. Within the supercritical water environment, hydrogen radicals acted as a source of hydrogen, leading to the saturation of final products, thereby yielding a high proportion of alkanes in the product oil from processing of low-density polyethylene.
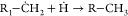


The increased concentration of aromatic compounds can be attributed to the high temperature and longer residence time used for the reaction. These were expected to favor the generation of more stable compounds, such as aromatic and cyclic compounds. This was due to the recondensation, aggregation, and polymerization processes that took place, resulting in the conversion of unstable oligomers into more stable aromatic compounds.^18^

Yan et al.^23^ proposed a degradation pathway for polyalkene plastics such as polyethylene and polypropylene using molecular dynamics modeling and density functional theory. They suggested that the polymer is initially degraded to produce oligomers, which are then further degraded into straight-chain alkanes and alkenes, promoted by thermal and supercritical water reactions. The branched polymeric structure of polypropylene also promotes cyclic reactions, producing cycloalkanes and cycloalkenes. They also suggested that the water-derived species, H^•^ and OH^•^, may also produce alcohols.

### 3.2 Supercritical Water Liquefaction of Mixed Plastics

The product yields from the binary mixtures of the plastics, composed of 1:1 mixtures of PP–PS, PP–LDPE, and LDPE–PS, and the ternary mixture of the plastics, composed of a 1:1:1 mixture of PP–LDPE–PS, obtained from the supercritical water liquefaction process are shown in Table 6. The reaction conditions were a plastic:water ratio of 1:4, a temperature of 450 °C, and a final pressure of 33 MPa with a residence time of 60 min. The results show that supercritical water liquefaction is an excellent option to process mixed plastic waste to acquire a high oil yield and a good conversion rate. It can also be noticed that co-liquefaction of plastic mixtures improved the yield from the plastic mixtures containing polypropylene and low-density polyethylene with higher oil yields than those obtained from the individual plastics. The results also showed that co-liquefaction of plastics in supercritical water significantly decreased the formation of solid residual char.

**Table 6:** Product Gas and Oil Yields (wt %) from the Supercritical Water Liquefaction of Different Mixtures of Plastics^a^

	plastic mixture			
	PP–PS	PP–LDPE	LDPE–PS	PP–LDPE–PS
gas yield	2.1	2.3	1.9	2.2
oil yield	96.9	97.0	97.2	96.4
solid	1.1	0.6	0.9	1.3
mass balance	100.1	99.9	100.0	99.9

a Process conditions: plastic:water ratio of 1:4, temperature of 450 °C, and final pressure of 33 MPa with a residence time of 60 min.

Zhao et al.^18^ reported that supercritical water liquefaction of a mixture of polyethylene and polypropylene showed interaction between the two polymers during the reaction, producing enhanced yields of oil. Seshasayee and Savage^19^ investigated the interaction of polypropylene, polystyrene, polyethylene terephthalate, and polycarbonate under supercritical water processing. They reported that the oil yield increased and displayed a positive synergy with the mixing of plastics. However, in contrast, the results from this work suggest that the oil yield is reduced due to interaction of the plastics compared to the oil yields from the individual plastics. The reason for this difference might be the difference in the heating rate and the decomposition characteristics of different plastic feedstocks with respect to those used by Zhao et al.^18^ and Seshasayee and Savage.^19^ For example, in another study by Seshasayee and Savage,^25^ they reported that supercritical water liquefaction of a mixture of polypropylene and polycarbonate yielded more oil on lowering the heating rate, whereas mixed polystyrene and PET showed no significant increase. The type and volume of the reactor used also influence the heating rate, thereby giving a different oil yield. According to Boel et al.,^6^ a higher heating rate might cause oil to surpass the optimal point before the heating process is completed, thereby converting more oil to gas and impacting the overall yield. Another reason for the difference in the oil yield might be the difference in the feedstock-to-water ratio used in the reaction. The present study used a 1:4 feedstock-to-water ratio, whereas the study by Seshasayee and Savage^19^ used a ratio of 1:8. The decomposition mechanisms are dependent on the amount of water, with less water facilitating free radical mechanisms rather than ionic mechanisms.^6^

**Figure 6:**
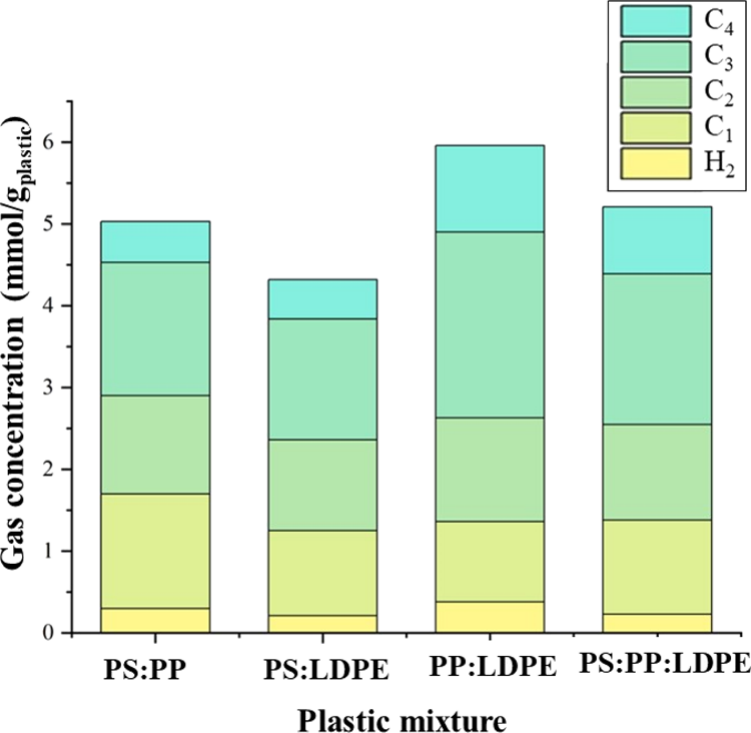
Gas composition from the supercritical water liquefaction of different mixtures of plastics. Process conditions were a plastic:water ratio of 1:4, a temperature of 450 °C, and a final pressure of 33 MPa with a residence time of 60 min.

Analysis of the product gases from the mixtures of the plastics was carried out, and the results are shown in Figure 6. The yield of the gaseous products can be attributed to the high temperature and pressure and residence time of the supercritical water process, but it also due to the interaction between the plastic feedstocks. Compared to the supercritical water liquefaction of polystyrene, the gas yield is higher when polystyrene is mixed with polypropylene, indicating that there exists an interaction between the plastics that influence the gas yield. The major components of all the gases produced included saturated hydrocarbons such as propane, ethane, methane, and butane. The C3 gas components were predominant among the products, with propane having the highest concentration, which may be mainly contributed from the polypropylene fraction of the mixture. It has been observed that increasing the reaction time and elevating the temperature result in a significant increase in propane concentration due to the hydrogenation of propene.^19^

Detailed analysis of the product oils obtained from the supercritical water liquefaction of the mixed plastics was undertaken using GC-MS/MS, and compositions of the oils from the different binary mixtures of PS–PP, PS–LDPE, and PP–LDPE are shown in the Supporting Information in Tables S1–S3, respectively. Also shown in the Supporting Information are the corresponding GC-MS/MS TICs of the product oils in Figures S1–S3.

**Figure 7:**
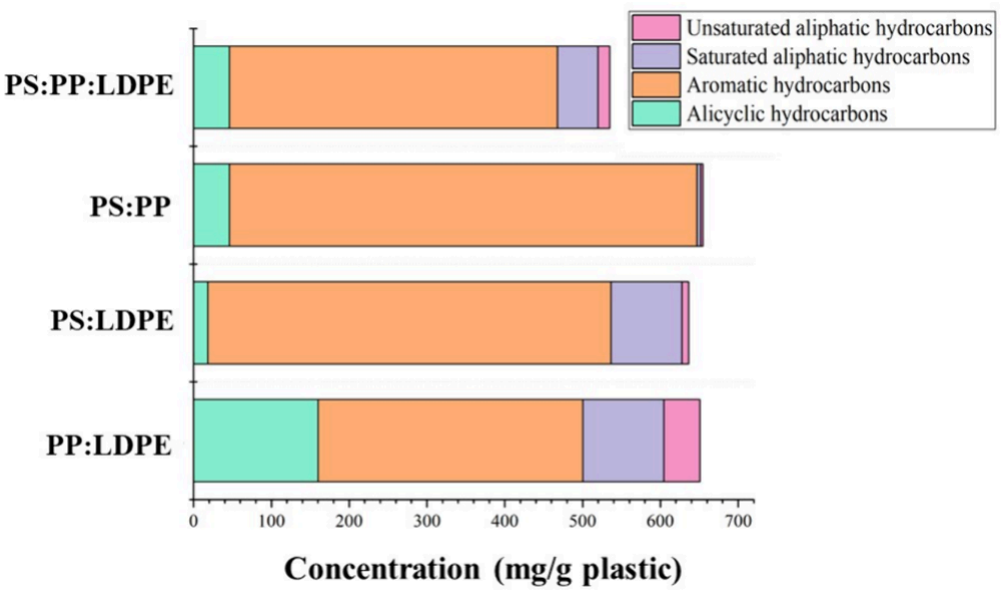
Composition of the product oils produced from the supercritical water liquefaction of different plastic mixtures. Process conditions: plastic:water ratio of 1:4, temperature of 450 °C, and final pressure of 33 MPa with a residence time of 60 min.

The oil components present in the binary mixtures of the plastics were classified into four groups: saturated aliphatic hydrocarbons, unsaturated aliphatic hydrocarbons, alicyclic hydrocarbons, and aromatic hydrocarbons. The results are shown in Figure 7. The analysis of oil produced by the co-liquefaction of polystyrene and polypropylene showed that aromatic hydrocarbons were the major component, comprising 76% of the GC-MS/MS total ion chromatogram (TIC) total peak area, followed by alicyclic hydrocarbons. This may be attributed to the cyclization and dehydrogenation reactions of the derived alkene liquefaction components during the supercritical water reaction process. Ethylbenzene, toluene, cumene, mesitylene, and *n*-propyl benzene were the major components, constituting about 57% of the TIC peak area. The co-liquefaction of polystyrene with low-density polyethylene also produced a high aromatic content of about 72% of the TIC peak area. The oil product also showed the presence of saturated hydrocarbons, which may be attributed to the contribution of the LDPE fraction. The major aliphatic components belonged to the carbon number range between C12 and C17. It can be noticed that the concentration of saturated hydrocarbons reduced significantly from 49% to 14% upon the addition of polystyrene to the plastic mixture, whereas aromatic hydrocarbons increased from 23% to 81%. This suggests that there is a positive synergy existing between polystyrene and low-density polyethylene. Similar to the oil produced from the PP–PS plastic mixture, the major constituents of the oil phase were toluene, ethylbenzene, and cumene.

Co-liquefaction of the mixed PP and LDPE plastics produced a product oil with aromatic hydrocarbons and alicyclic hydrocarbons as the major components. It was observed that the alkane content decreased from 48% to 16% upon adding polypropylene to LDPE. Zhao et al.^18^ also reported that the concentration of cyclic components increased significantly in the product oil produced from the supercritical water liquefaction of plastic mixtures of PE and PP. They suggested that cyclization was significantly promoted during the co-liquefaction of the plastic mixture in supercritical water and that adding PP was advantageous in enhancing the production of cyclic hydrocarbons. They concluded that reactions were primarily promoted by the formation of oligomers and free radicals through the degradation of both PP and PE, which readily underwent cyclization. Similarly, Zhao et al.^18^ also showed that there was an increase in the alkene content, indicating the notable influence of polypropylene on the composition of the oil phase.

The tertiary plastic mixture of PS, PP, and LDPE in the ratio of 1:1:1 also produced a high oil yield. Aromatic hydrocarbons formed the majority of the oil composition, constituting about 66% of the GC-MS/MS TIC peak area. The high concentration of ethylbenzene, toluene, cumene, and xylene can be attributed to the presence of polystyrene and polypropylene as well as the high reaction temperature and residence time. The substantial decrease in the concentration of saturated hydrocarbons and unsaturated aliphatic hydrocarbons compared to the results of PP–LDPE shows that the presence of polystyrene further facilitated the production of aromatics while decreasing the yield of alkenes and alkanes. Similarly, the incorporation of PP promoted the production of cyclic compounds and suppressed the production of alkanes.

Table 7 shows the main compounds identified in the product oils from the tertiary mixture of the three plastics PS, PP, and LDPE. The total peak area of identified compounds in the product oil represents 79.06% of the oil compounds. The remaining 20.94% represents low concentration compounds. The detailed analysis of the oils reflects the contribution of compounds from the individual plastics, that is, a mixture of aromatic compounds was mainly from the pyrolysis of polystyrene, aliphatic compounds (particularly alkanes and alkenes) were mainly from low-density polyethylene, and cyclic alkanes and alkenes and alkylated benzenes were mainly from polypropylene.

**Table 7:** Compounds Identified by GC-MS/MS in the Product Oil from the Supercritical Water Liquefaction of Tertiary Mixture of Polystyrene, Polypropylene, and Low-Density Polyethylene at Conditions of 450 °C and Final Pressure of 33 MPa with a Residence Time of 60 min and Plastic:Water Ratio of 1:4^a^

retention time (min)	% of total area	compound	concentration (mg g^–1^ of PS/PP/LDPE)
2.084	0.602	cyclohexane	3.84
2.714	0.719	benzene	4.58
3.208	1.015	3-methylhexane	6.47
3.696	0.761	1-ethyl-1-methylcyclopentane	4.85
4.727	15.675	toluene	99.93
5.558	0.565	cyclohexane, 1-ethyl-2-methyl	3.60
5.876	1.702	3-ethylhexane	10.85
6.129	0.594	1,4-pentadiene, 2,3,4-trimet	3.79
6.29	0.629	cyclopentane, 1,1,3,4-tetram	4.01
7.224	2.121	cyclohexane, 1,1,3-trimethyl	13.52
8.382	1.521	1,1,4-trimethylcyclohexane	9.70
8.542	18.362	ethylbenzene	117.06
8.997	2.627	*o*-xylene	16.75
9.075	0.948	*p*-xylene	6.04
10.196	1.008	ethylbenzene	6.43
10.894	1.404	decane, 2,5,6-trimethyl-	8.95
12.11	5.86	benzene, (1-methylethyl)-	37.36
14.012	2.472	benzene, propyl-	15.76
14.525	0.998	benzene, 1-ethyl-3-methyl-	6.36
15.096	2.645	benzene, 1,2,4-trimethyl-	16.86
16.725	1.395	benzene, 1,3,5-trimethyl-	8.89
17.551	1.038	decane C10	6.62
21.069	1.408	undecane C11	8.98
22.613	0.745	2,4-dimethylstyrene	4.75
23.78	1.068	dodecane C12	6.81
26.08	0.55	benzene, pentyl-	3.51
27.009	0.901	naphthalene	5.74
27.906	1.013	tridecane C13	6.46
30.87	1	naphthalene, 1,2,3,4-tetrahy	6.38
31.185	0.875	tetradecane C14	5.58
31.329	0.948	2-methylnaphthalene	6.04
34.04	0.762	pentadecane C15	4.86
36.191	0.721	1,1′-biphenyl, 4-methyl-	4.60
36.632	0.798	hexadecane C16	5.09
39.044	0.718	heptadecane C17	4.58
41.309	0.782	octadecane C18	4.99
43.458	0.76	nonadecane C19	4.85
45.499	0.566	eicosane C20	3.61
46.868	0.786	phenylnaphthalene	5.01
total	79.06		504.02

a Listed are the highest concentration compounds identified in the oil.

Zhao et al.^18^ have suggested a reaction mechanism to explain the interaction between different aliphatic polymers such as polyethylene and polypropylene under supercritical water conditions. They proposed that the aliphatic polymers are initially degraded via random polymer bond scission to produce long-chain aliphatic oligomers (alkanes and alkenes). At more intense process conditions, the oligomers are further depolymerized by bond scission to produce lower molecular weight alkane and alkene hydrocarbons. Cyclic and aromatic compounds may also be formed from the cyclization and aromatization of the aliphatic hydrocarbon fragments; the presence of branched chains in the polypropylene structure facilitates these reactions. It has been proposed by Yan et al.^23^ that the reaction mechanism for the interaction between aromatic and aliphatic polymers such as polyethylene and polypropylene with polystyrene involves interaction of fragments formed from polymer decomposition under supercritical water conditions. For example, C_2_H_2_ fragments from polypropylene or polyethylene react with monoaromatic or polycyclic aromatic fragments to produce new oil fragments. Seshasayee and Savage^19^ also suggest interaction of reactive fragment species that aid polymer decomposition in the supercritical water liquefaction process. In particular, the key role of polystyrene was suggested, whereby fragments from other polymers promote polystyrene decomposition at lower temperatures and thereby produce more reactive species from polystyrene, thus facilitating further polymer decomposition and interaction.

### 3.3 Synergistic Interactions of the Plastics

To further understand the synergistic interactions of the different plastics for the supercritical water reactions, a “synergy factor” was calculated. The synergy factor was determined using eqs 1 and 2 in relation to the product oil, gas, and solid, eqs 3 and 4 for the gas composition, and eqs 5 and 6 for the oil composition (modified from Mukundan et al.).^42^
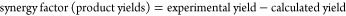
1The calculated yield of each reaction product was obtained using eq 2 based on their yields from the of the individual feedstock.
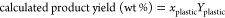
2where *x* = mass fraction in mixture, *Y* = wt % yield, and “plastic” = PS, PP, or LDPE.
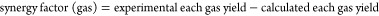
3The calculated gas yield for each component product gas was obtained using eq 4 for gases from both the individual and mixed plastics obtained from the supercritical water liquefaction experiments.
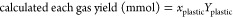
4where *x* = mass fraction and *Y* = mmol/g of each gas from PS, PP, or LDPE.
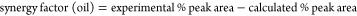
5The calculated % peak area of each compound class was obtained using eq 6 for oils from both the individual and mixed plastics obtained from the supercritical water liquefaction experiments.
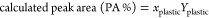
6where *x* = mass fraction and *Y* = peak area % of each compound class in the PS, PP, or LDPE oils.

**Figure 8:**
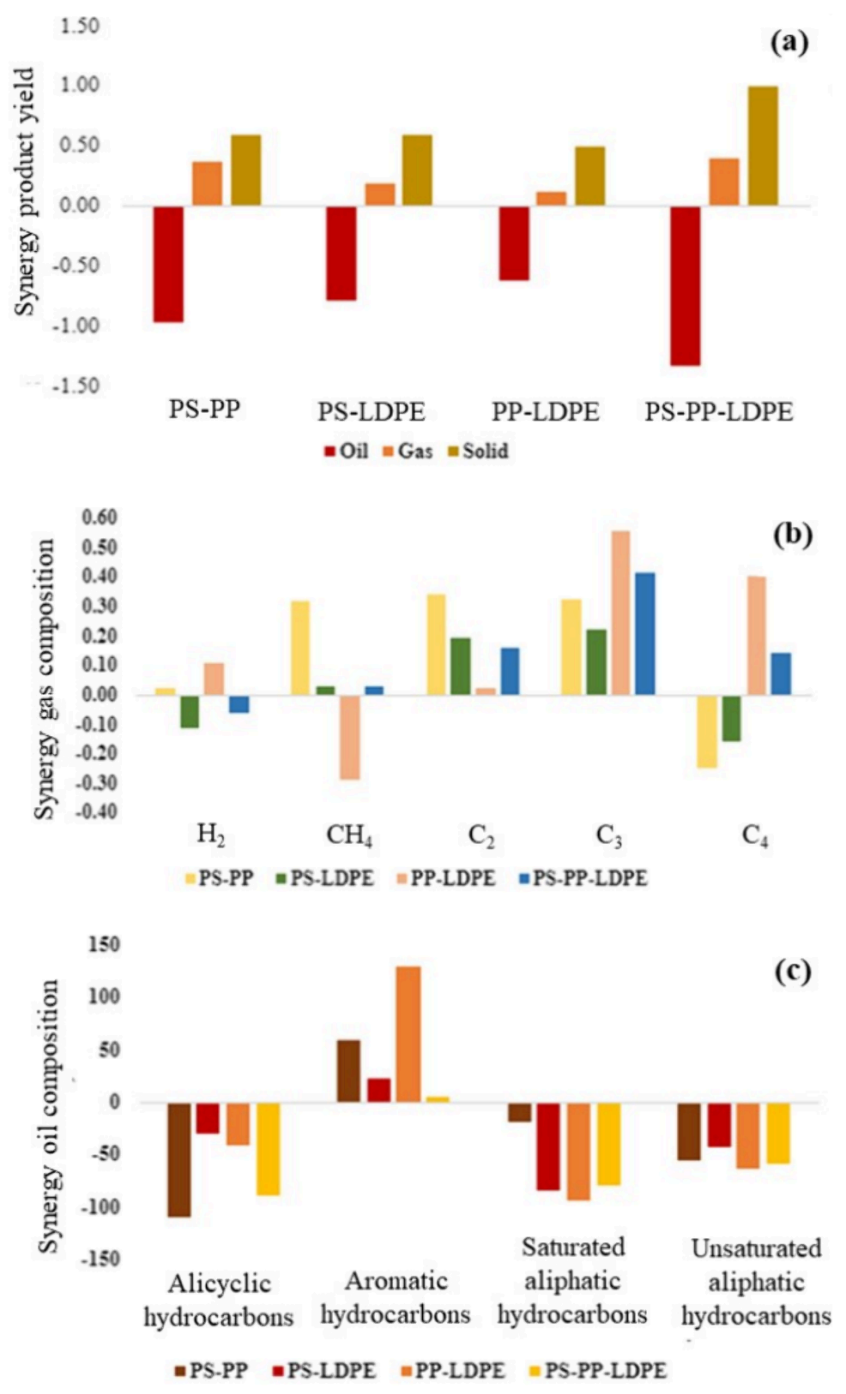
Synergy factors for the interaction of the binary and ternary mixtures of the PS, PP, and LDPE plastics in relation to (a) the product yield, (b) gas composition, and (c) oil composition.

The determination of the synergistic interaction between the binary and ternary mixtures of the PS, PP, and LDPE plastics in relation to the product yields of oil, gas, and solid based on eqs 1 and 2 is shown in Figure 8a. The co-liquefaction of plastics such as polystyrene, polypropylene, and low-density polyethylene in a 1:1 ratio shows different synergic effects with respect to the products formed. The values of synergy factors in Figure 8a suggest an indication of synergistic interaction resulting in a reduced oil yield, a higher solid yield, and a higher gas yield. The interactions between the plastics promoted both gas and char formation, with negative synergies for oil production.

Figure 8b shows the synergy factors for the interaction of the binary and ternary mixtures of the PS, PP, and LDPE plastics in relation to the gas composition (eqs 3 and 4). The three binary mixtures of the plastics (PS–PP, PS–LDPE, and PP–LDPE) and the ternary plastic mixture (PS–PP–LDPE) all showed a positive synergy factor for the production of C2 and C3 hydrocarbons. The PS–PP plastic mixture also produced a positive synergy factor for C1 production but was reduced for C4 production. The addition of polystyrene suppresses the production of C4 gas components. The synergistic effect for methane is found to be generally positive for all mixture combinations except for the mixture of polyalkene plastics (PP–LDPE).

Figure 8c shows the synergy factors for the composition of the product oil for the interaction of the binary mixtures of the PS, PP, and LDPE plastics and the ternary mixture of the three plastics (eqs 5 and 6). There is a clear synergy for the interaction of all of the plastics that produces a positive factor for aromatic hydrocarbon production. Noticeably, the synergy factors for the alicyclic hydrocarbons, saturated aliphatic hydrocarbons, and unsaturated aliphatic hydrocarbons are all negative, suggesting interaction of these species to produce aromatic hydrocarbons. It can be observed that the alkane content was decreased when LDPE was mixed with PP and/or PS, which corresponds to the study conducted by Zhao et al.,^18^ which involved the supercritical water co-liquefaction of LLDPE and PP. Similarly, the alkene content and alicyclic compound content decreased in the plastic mixtures even though oil from PP had a higher content of alicyclic and alkene compounds, giving lower experimental values than the theoretical value and thereby indicating negative synergy.

Plastic pollution has become a critical environmental issue, necessitating urgent action and innovative solutions in relation to its management. To address this challenge, the supercritical water liquefaction of plastic waste offers a promising and sustainable approach. The process involves subjecting plastic waste to high-temperature and high-pressure conditions in supercritical water, breaking down complex polymers into smaller molecules. The results of this research highlight the effectiveness of supercritical water in converting plastic waste, including mixed plastics, to valuable liquid oil products. These results show that supercritical water liquefaction is an excellent method to produce high yields of oil at over 97 wt % that is suitable for use as a liquid fuel. Also, high yields of aromatic compounds are also produced with potential applications in various industries. The gaseous components such as C1–C4 hydrocarbons have a high calorific value and could be used to provide the energy requirements for the process.

## 4 Conclusions

This work has investigated the supercritical water liquefaction of common plastic wastes, such as low-density polyethylene, polypropylene, and polystyrene as well as their mixtures. The reactions were carried out for different feedstock-to-water ratios, i.e., 1:3,1:4,1:6, and 1:9, at a temperature of 450 °C and pressures between 22 and 33 MPa with a residence time of 60 min. The products were mainly composed of oil and gas. The liquefaction of the plastics in the presence of supercritical water was found to suppress the formation of carbonaceous residues and enhance the oil yield. The gas phase mainly consisted of light hydrocarbons such as methane, ethane, propane, and butane, with propane found to be the most abundant gas component. High yields of oil of ∼97 wt % were obtained from the supercritical water liquefaction of plastics without the presence of catalysts. The oil phase contained a mixture of alkanes, alkenes, cyclic hydrocarbons, and aromatic hydrocarbons. The aromatic hydrocarbons and alicyclic hydrocarbons were the major products in the product oil from the supercritical water liquefaction of polystyrene and polypropylene, whereas alkanes were predominant in the oil obtained from LDPE. The GC-MS/MS analysis of the oil obtained from binary (1:1) and tertiary (1:1:1) plastic mixtures showed it exhibited aromatic hydrocarbons as the major constituent, indicating synergistic interaction between the plastic types. It was found that the incorporation of PP in the mixture facilitated the production of cyclic compounds and suppressed the production of alkanes.

## Supporting Information Available

GC-MS/MS compositional analysis tables and total ion chromatograms of the oils produced from the binary plastic mixtures (PDF)
