# Supplementary material for: Supercritical Water Liquefaction of Mixed Waste Polystyrene, Polypropylene, and Polyethylene for Production of High Yield Oils
Source: Energy Fuels. 2024 Jul 3;38(14):12810–23. doi: 10.1021/acs.energyfuels.4c01819 (PMC11264203; doi:10.1021/acs.energyfuels.4c01819)
Supplement: Supplementary file 1 — ef4c01819_si_001.pdf [file ef4c01819_si_001.pdf]

Supercritical water liquefaction of mixed waste polystyrene, polypropylene and polyethylene for production of high yield oils

Maria Mathew, Mohamad A. Nahil, Andrew B. Ross, Paul T. Williams *
[1]School of Chemical and Process Engineering, University of Leeds, Leeds LS2 9JT, UK

(*Corresponding author; Tel: #44 1133432504; Email: p.t.williams@leeds.ac.uk)

SUPPLEMENTARY INFORMATION

Table S1. Compounds identified by GC-MS/MS in the product oil from the supercritical water liquefaction of mixed mixture of polystyrene and polypropylene at conditions of temperature, 450 °C and final pressure of 33 MPa, with a residence time of 60 mins and plastic: water ratio of 1:4. Listed are the thirty highest concentration compounds identified in the oil.

Retention Time (min.)	Peak area (%)	Compound	Concentration (mg g^{-1} of PS/PP)
2.72	0.502	Benzene	3.36
4.752	16.242	Toluene	3.95
6.324	0.595	Cyclopentane, 1,1,3,4-tetramethyl	2.00
7.257	2.365	Cyclohexane, 1,1,3-trimethyl	2.42
8.424	1.602	1,1,4-trimethylcyclohexane	1.80
8.594	22.815	Ethylbenzene	127.91
9.041	2.617	p-xylene	1.65
9.12	0.947	Benzene, 1,3-dimethyl-	4.69
10.242	0.606	p-xylene	2.71
12.167	7.875	Benzene, (1-methylethyl)-	18.62
14.071	2.831	Benzene, propyl-	3.17
14.585	0.702	Benzene, 1-ethyl-3-methyl-	12.62
15.159	4.218	Benzene, 1,2,3-trimethyl-	179.67
16.785	1.529	Benzene, 1,2,4-trimethyl-	20.61
21.127	1.295	Benzene, butyl-	7.46
21.239	0.579	Benzene, 2-ethyl-1,4-dimethyl	0.74
22.663	0.518	2,4-dimethylstyrene	2.39
23.549	0.591	Benzene, (1,2-dimethylpropyl	4.77
26.122	0.516	Benzene, pentyl-	62.02
27.049	1.167	Naphthalene	22.29
27.233	0.448	1H-Indene,2,3-dihydro-2,2-di	5.53
30.905	1.361	2 methylnaphthalene	33.22
31.365	0.994	Benzocycloheptatriene/1-methyl naphthalene	2.09
34.473	0.677	Naphthalene, 1,7-dimethyl-	12.04

34.814	0.974	Diphenylmethane	10.20
36.227	0.93	1,1'-Biphenyl, 4-methyl-	4.56
36.258	0.644	Benzene, 1,1'-ethylidenebis-	4.08
36.469	0.5	2-Ethyl-1-dodecanol	4.65
37.088	1.315	Bibenzyl	4.06
38.698	1.042	4,4'-dimethylbiphenyl	9.19
46.903	0.71	2-phenylnaphthalene	3.53
**Total**	**79.71**		**577.99**

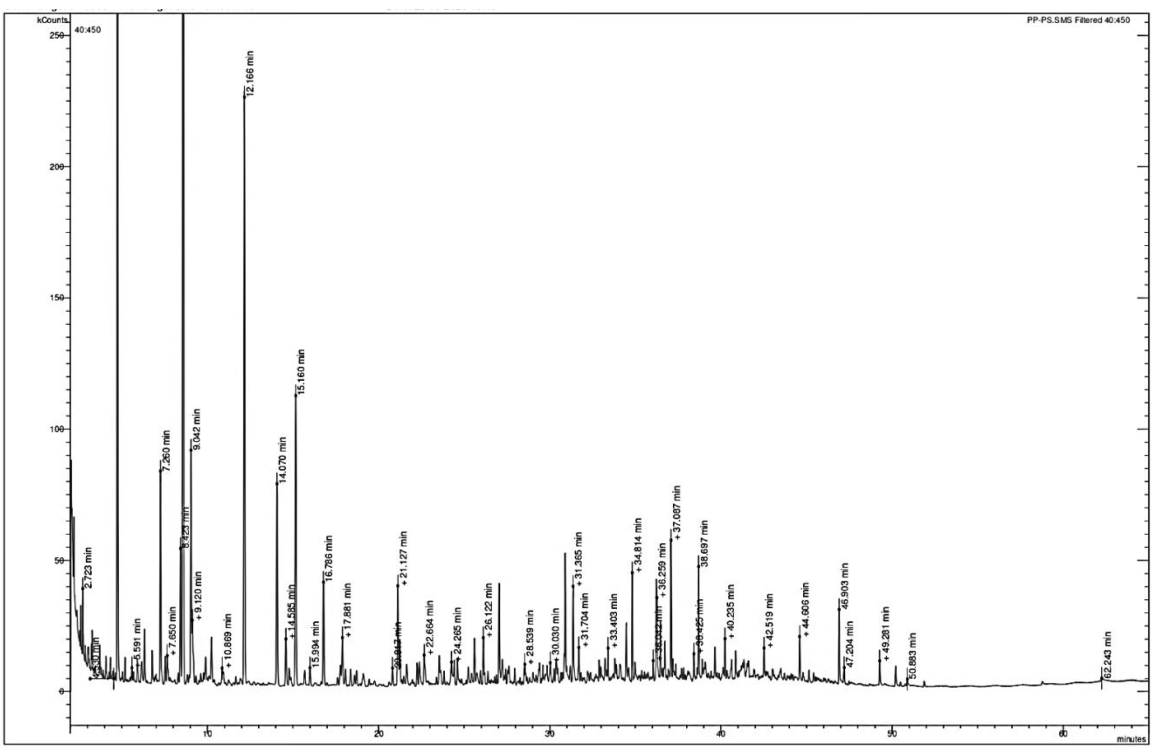

Figure S1. GC-MS/MS total ion chromatograms for oils derived from the supercritical water liquefaction of mixed polystyrene and polypropylene at conditions of 450 °C and final pressure of 33 MPa, with a residence time of 60 mins and plastic: water ratio of 1:4.

Table S2. Compounds identified by GC-MS/MS in the product oil from the supercritical water liquefaction of mixed mixture of polystyrene and low density polyethylene at conditions of temperature, 450 °C and final pressure of 33 MPa, with a residence time of 60 mins and plastic: water ratio of 1:4. Listed are the thirty highest concentration compounds identified in the oil.

Retention Time (min.)	Peak area (%)	Compound	Concentration ($mg\ g^{-1}$ of PS/LDPE)
2.093	0.846	Cyclohexane	6.05
2.668	1.429	benzene	10.21
3.162	1.126	3-methyl hexane	8.05
3.645	0.637	Cyclopentane 1,1,3-trimethyl/	4.55
4.664	24.145	Toluene	172.54
5.808	1.048	3-ethyl hexane	7.49
8.469	21.491	Ethyl benzene	153.57
8.912	0.978	o-xylene	6.99
10.108	0.801	m-Xylene	5.72
10.803	0.704	Hexane, 2,4-dimethyl-	5.03
12.019	6.443	Benzene, (1-methylethyl)-	46.04
13.915	2.338	Benzene, propyl-	16.71
14.427	0.567	Benzene, 1-ethyl-3-methyl-	4.05
17.451	0.781	C10 alkane	5.58
20.976	1.019	C11 Undecane	7.28
22.53	0.66	Benzene, 1-butenyl-, (E)-	4.72
23.71	1.026	C12 dodecane	7.33
26.016	0.644	Benzene, pentyl-	4.60
26.943	1.072	Naphthalene	7.66
27.85	1.036	C13 tridecane	7.40
30.811	0.701	2 methylnaphthalene	5.01
31.133	0.974	C14 tetradecane	6.96
31.271	1.048	1H-Indene, 1-ethylidene-	7.49
33.989	0.963	C15 pentadecane	6.88
34.726	0.79	Diphenylmethane	5.65
36.383	0.625	1,1'-Biphenyl, 4-methyl-	4.47
36.583	1.294	C16 hexadecane	9.25
37.002	1.262	Bibenzyl	9.02
38.994	1.11	C17 heptadecane	7.93
41.26	1.238	C18 octadecane	8.85
43.406	1.043	C19 nonadecane	7.45
45.446	0.547	C20 eicosane	3.91
50.108	0.52	m terphenyl	3.72
**Total**	**80.91**		**578.14**

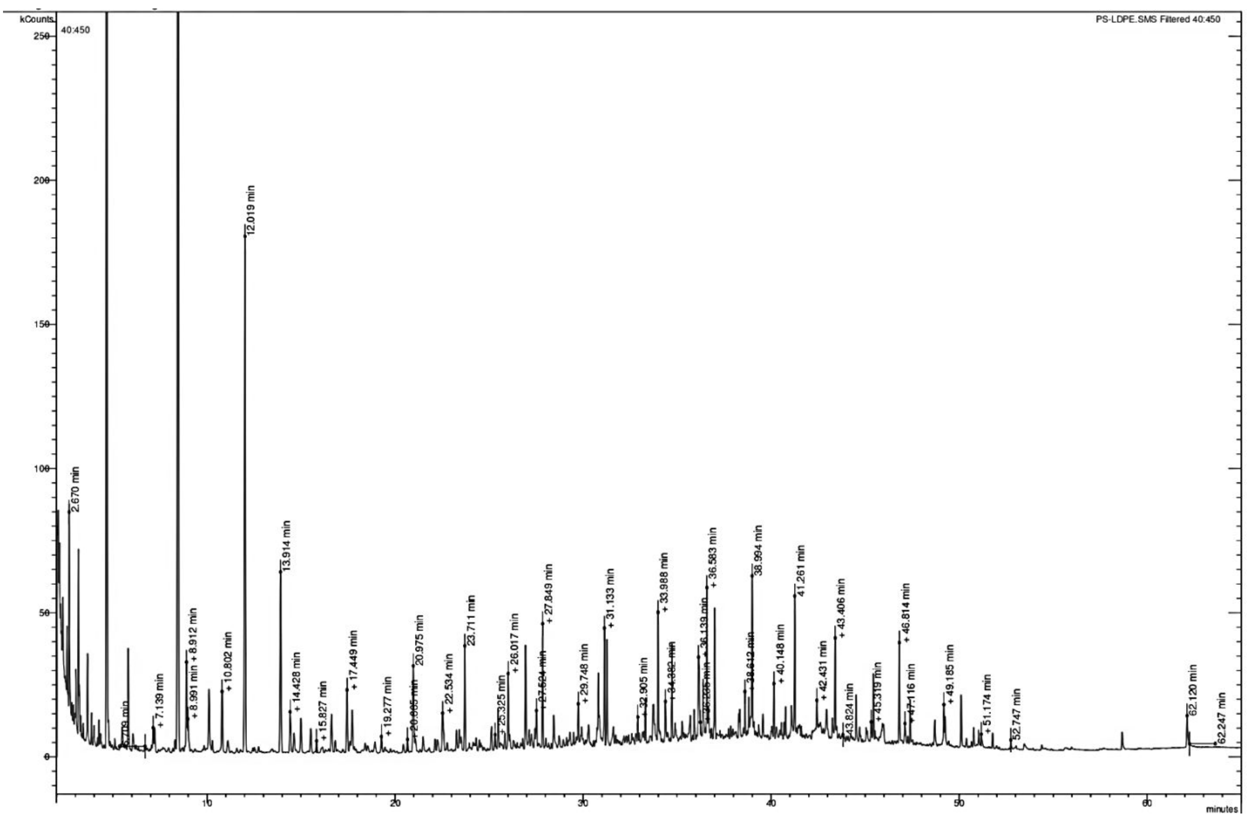

Figure S2. GC-MS/MS total ion chromatograms for oils derived from the supercritical water liquefaction of mixed polystyrene and low density polyethylene at conditions of 450 °C and final pressure of 33 MPa, with a residence time of 60 mins and plastic: water ratio of 1:4.

Table S3. Compounds identified by GC-MS/MS in the product oil from the supercritical water liquefaction of mixed mixture of polypropylene and low density polyethylene at conditions of temperature, 450 °C and final pressure of 33 MPa, with a residence time of 60 mins and plastic: water ratio of 1:4. Listed are the thirty highest concentration compounds identified in the oil.

Retention Time (min.)	Peak area (%)	Compound	Concentration (mg g^{-1} of PP/LDPE)
2.08	3.67	Cyclohexane	25.00
2.17	1.37	2-pentene, 3-methyl	9.33
2.35	1.65	Cyclopentane methyl	11.26
2.56	1.05	2-Pentene 2,4-dimethyl	7.18
2.69	2.21	benzene	15.08
2.82	0.84	3,7-dimethyl 1-octene	5.75
2.97	0.85	3-methyl octene/1,3 dimethyl cyclopentane	5.78
3.00	0.67	1,3-dimethyl cyclopentane	4.57
3.04	1.11	1,3-dimethyl cyclopentane	7.57
3.17	4.10	3-methyl hexane	27.92
3.22	0.44	1,3-Pentadiene 2,3-dimethyl	3.00
3.65	3.39	methyl cyclohexane/1-ethyl-1-methyl cyclopentane	23.07
3.86	0.75	Cyclopentane butyl	5.12
4.00	1.16	3-methylene heptane	7.88
4.26	0.93	3-methyl 2,4-hexadiene	6.34
4.66	9.36	toluene	63.78
4.76	0.43	4-methyl1,4- hexadiene	2.93
5.10	1.07	cyclohexane 1-ethyl-2-methyl	7.28
5.51	1.35	Cyclopentane 1-ethyl-2-methyl	9.22
5.59	0.50	2,4-hexadiene 2,5-dimethyl	3.43
5.81	3.47	3-ethyl hexane	23.67
6.06	1.51	Cyclopentane 1,1-ethylidine bis	10.27
6.22	1.44	Cyclopentane, 1,1,3,4-tetramethyl	9.84
7.15	3.30	Cyclohexane, 1,3,5-trimethyl	22.45
7.42	0.66	1,1,3-trimethyl cyclohexane	4.47
8.30	2.94	Cyclohexane, 1-ethyl-1,3-dimethyl	20.04
8.44	2.35	Ethylbenzene	16.04
8.92	8.40	p-xylene	57.22
8.99	2.80	m-Xylene	19.10
10.11	3.00	o-xylene	20.46
10.80	1.73	Hexane, 2,4-dimethyl-	11.77
14.42	3.09	Benzene, 1-ethyl-2-methyl-	21.08
14.62	1.17	1-ethyl,3-methyl benzene	7.99
14.99	4.75	Benzene, 1,2,3-trimethyl-	32.33
16.61	4.29	Benzene, 1,2,4-trimethyl-	29.23
17.44	1.45	C10 Decane	9.86

21.08	1.10	C11 undecane	7.47
23.70	1.29	C12 dodecane	8.75
25.12	1.14	Benzene 4-ethenyl-1,2-dimethyl	7.79
25.48	1.42	2,4-Dimethylstyrene	9.65
27.11	1.24	Naphthalene	8.45
27.42	0.69	Benzene 1-methyl-3-(1-methyl-2-propenyl	4.69
27.83	1.04	C13 tridecane	7.05
30.80	0.87	2 methylnaphthalene	5.89
31.12	0.76	C14 tetradecane	5.14
31.26	0.79	1H-Indene, 1-ethylidene-	5.41
33.98	0.61	Naphthalene 1,2-dihydro 1,5,8-trimethyl	4.14
34.37	0.65	C15 pentadecane	4.44
36.57	0.40	C16 hexadecane	2.70
**Total**	**95.25**		**648.86**

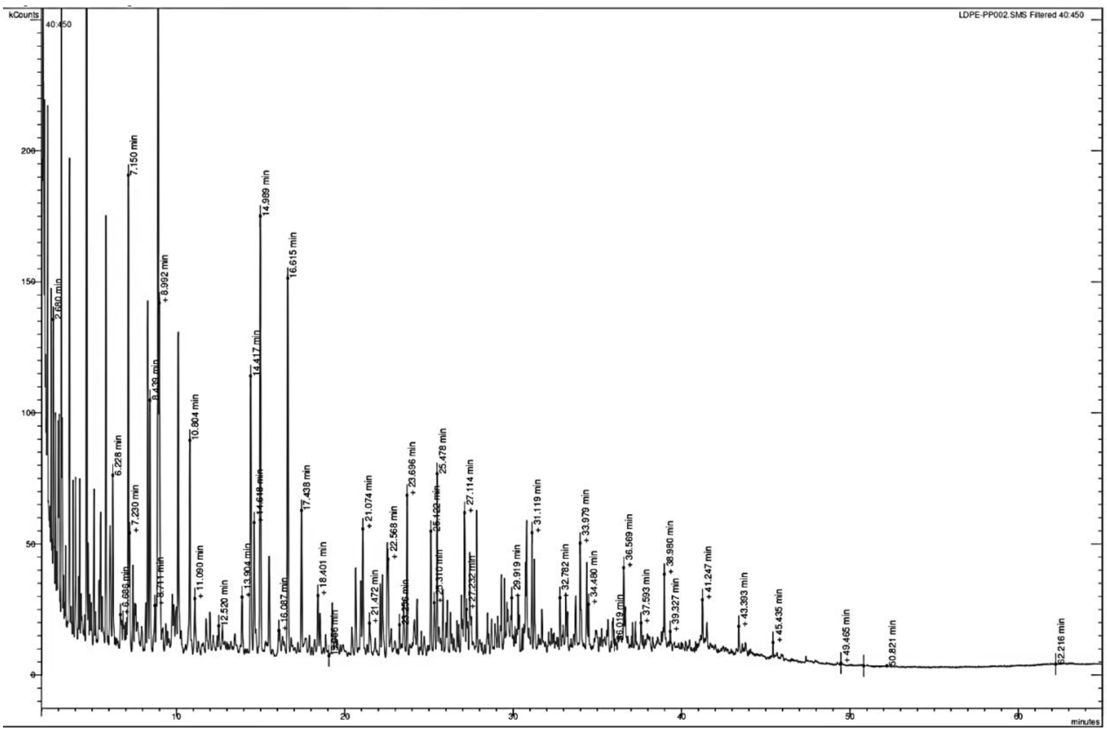

Figure S3. GC-MS/MS total ion chromatograms for oils derived from the supercritical water liquefaction of mixed polypropylene and low density polyethylene at conditions of 450 °C and final pressure of 33 MPa, with a residence time of 60 mins and plastic: water ratio of 1:4.
